# A multiple gene complex on rice chromosome 4 is involved in durable resistance to rice blast

**DOI:** 10.1007/s00122-012-1852-4

**Published:** 2012-03-25

**Authors:** S. Fukuoka, R. Mizobuchi, N. Saka, S. Ivan, T. Matsumoto, K. Okuno, M. Yano

**Affiliations:** 1 National Institute of Agrobiological Sciences, Kannondai 2-1-2, Tsukuba, Ibaraki 305-8602 Japan; 2 Present Address: Graduate School of Life and Environmental Sciences, University of Tsukuba, Tennohdai 1-1-1, Tsukuba, Ibaraki 305-8572 Japan; 3 Aichi Agricultural Research Center, Mountainous Region Institute, Innahashi, Toyota, Aichi 441-2513 Japan; 4 Present Address: Aichi Agricultural Research Center, Ikeura, Anjo, Aichi 446-0066 Japan; 5 All Russian Rice Research Institute, Bolozerniy, Krasnodar, 350921 Russia

## Abstract

Quantitative trait loci (QTLs) for resistance to rice blast offer a potential source of durable disease resistance in rice. However, few QTLs have been validated in progeny testing, on account of their small phenotypic effects. To understand the genetic basis for QTL-mediated resistance to blast, we dissected a resistance QTL, *qBR4*-*2*, using advanced backcross progeny derived from a chromosome segment substitution line in which a 30- to 34-Mb region of chromosome 4 from the resistant cultivar Owarihatamochi was substituted into the genetic background of the highly susceptible Aichiasahi. The analysis resolved *qBR4*-*2* into three loci, designated *qBR4*-*2a*, *qBR4*-*2b*, and *qBR4*-*2c*. The sequences of *qBR4*-*2a* and *qBR4*-*2b*, which lie 181 kb apart from each other and measure, 113 and 32 kb, respectively, appear to encode proteins with a putative nucleotide-binding site (NBS) and leucine-rich repeats (LRRs). Sequence analysis of the donor allele of *qBR4*-*2a*, the region with the largest effect among the three, revealed sequence variations in the NBS-LRR region. The effect of *qBR4*-*2c* was smallest among the three, but its combination with the donor alleles of *qBR4*-*2a* and *qBR4*-*2b* significantly enhanced blast resistance. *qBR4*-*2* comprises three tightly linked QTLs that control blast resistance in a complex manner, and thus gene pyramiding or haplotype selection is the recommended strategy for improving QTL-mediated resistance to blast disease through the use of this chromosomal region.

**Electronic supplementary material:**

The online version of this article (doi:10.1007/s00122-012-1852-4) contains supplementary material, which is available to authorized users.

## Introduction

Rice blast, a destructive disease of rice, is caused by the fungal pathogen *Magnaporthe oryzae* (Ou 1985). To date, more than 80 genes for blast resistance have been recorded, of which 60 are genetically mapped (Gramene database: http://www.gramene.org/). Most are race specific and are characterized by a hypersensitive reaction (Greenberg and Yao 2004). However, genes for race-specific resistance are rapidly been overcome by the pathogen (Bonman et al. 1992; Kiyosawa 1982) and so cannot support sustainable crop production.

In contrast to race-specific resistance, resistance controlled by quantitative trait loci (QTLs) is characterized by a susceptible infection type, usually without race specificity or gene-for-gene interaction (Ezuka 1972; Parlevliet 1979). In general, cultivars carrying resistance QTLs have maintained their resistance for a long time, possibly because of decreased selection pressure against the pathogen. Therefore, the discovery of QTLs in resistant cultivars is crucial to our understanding of the genetic control of QTL-mediated blast resistance (Huang et al. 2011; Jia and Liu 2011; Liu et al. 2011; Shi et al. 2010).

Japanese upland rice cultivars are potential donors of QTL-mediated resistance (Abe et al. 1976). Their resistance is controlled by multiple genes (Fukuoka and Okuno 2001; Higashi and Kushibuchi 1978; Kato et al. 2002; Miyamoto et al. 2001). For example, the resistance QTLs identified in cultivar Owarihatamochi have been detected in three regions, of which *pi21*, on chromosome 4, explains 45.7% of the phenotypic variation (Fukuoka and Okuno 2001). Although extensive efforts have been made to introduce blast resistance from upland cultivars into elite irrigated cultivars, substantial QTL-mediated resistance has not been introduced, on account of poor eating quality and low yield potential caused by linkage drag (Higashi 1995; Morimoto 1980; Saka 2006). Recently, *pi21* has been cloned by map-based cloning, allowing us to solve the long-term problem of linkage drag (Fukuoka et al. 2009). This example is a clear demonstration of the value of validation and fine mapping of QTLs for blast resistance.

The other two QTLs in Owarihatamochi, on chromosomes 4 (*qBR4*-*2*) and 12 (*qBR12*-*1*), explain 29.4 and 13.7%, respectively, of the phenotypic variation (Fukuoka and Okuno 2001). As *pi21* confers moderate resistance, the resistance of elite cultivars carrying *pi21* can be enhanced by combination with other resistance QTLs. To increase the set of genes for QTL-mediated resistance, extensive genetic studies have investigated QTLs with different magnitudes of effects from several cultivars (Fukuoka and Okuno 2001; Fukuoka et al. 2009; Huang et al. 2011; Jia and Liu 2011; Kato et al. 2002; Liu et al. 2011; Miyamoto et al. 2001; Nguyen et al. 2006; Shi et al. 2010; Suh et al. 2009; Terashima et al. 2008; Wang et al. 1994; Xu et al. 2008; Zenbayashi et al. 2002; Zenbayashi-Sawata et al. 2007). Although the elimination of undesirable characters closely linked to loci of interest is a key factor in the successful transfer of genes to commercial cultivars (Fukuoka et al. 2009), most QTLs have not been delimited in advanced progeny lines. Our aim here was to analyze *qBR4*-*2*, with the second largest effect after *pi21*, in order to map it precisely by fine genetic analysis using lines derived from a chromosome segment substitution line (CSSL) carrying this QTL.

## Materials and methods

### Plant materials

**Fig. 1:**
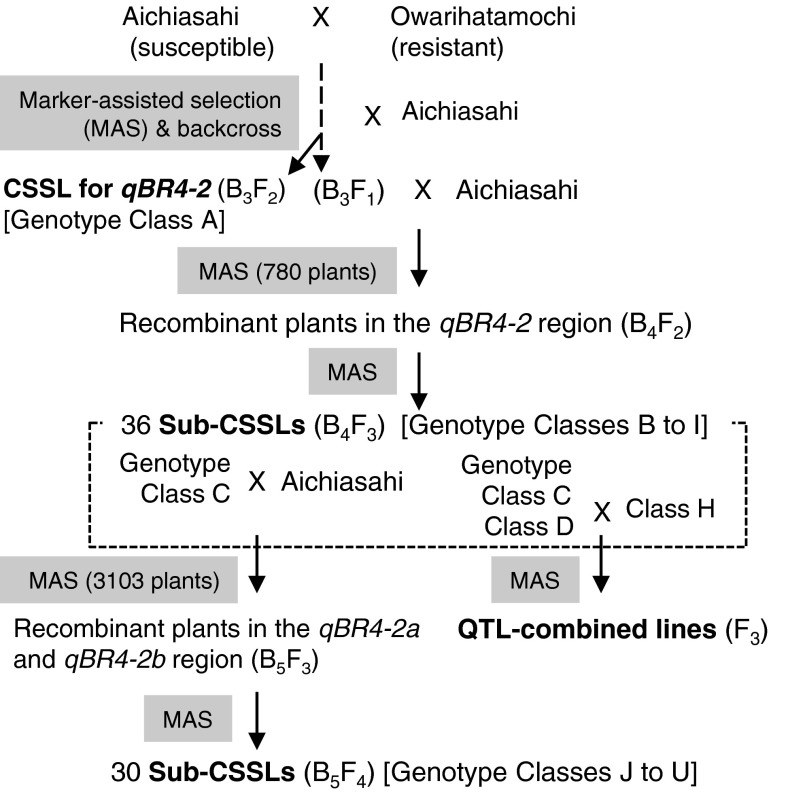
Development of the plant materials used in the present study

Their highly homogeneous genetic background makes CSSLs suitable for identifying QTLs under complicated genetic control in several crop species, including rice (Ali et al. 2010; Ebitani et al. 2005; Eshed and Zamir 1995; Hirabayashi et al. 2010; Howell et al. 1996; Kubo et al. 2002; Xu et al. 2010; Yoshimura et al. 2010). Using three rounds of backcrossing and marker-assisted selection, we developed a CSSL in which the 30- to 34-Mb region of chromosome 4 between the DNA marker loci *RM317* and *C1016* from the resistant upland rice cultivar Owarihatamochi was substituted into the genetic background of the susceptible lowland cultivar Aichiasahi (Fig. 1). Owarihatamochi has a high level of resistance controlled by multiple QTLs, whereas Aichiasahi which carries the race-specific genes *Pia* and *Pi19*(*t*) (Koide et al. 2011) is highly susceptible to blast under field condition. The rest of the genome was homogeneous for Aichiasahi, as confirmed by the DNA markers used in our previous study (Fukuoka and Okuno 2001).

**Fig. 2:**
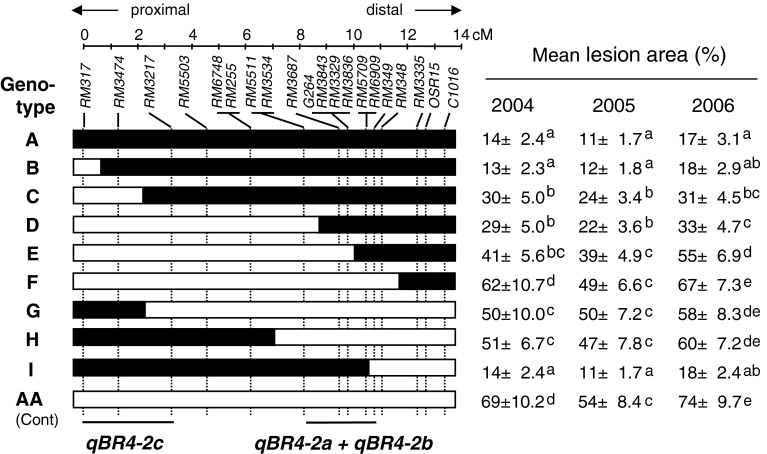
Genetic linkage map and graphical genotypes of sub-CSSLs around *qBR4*-*2*. *Black bars* indicate chromosome regions derived from the resistant Owarihatamochi; *white bars* indicate those derived from the susceptible Aichiasahi. The locations of *qBR4*-*2a*/*qBR4*-*2b* and *qBR4*-*2c*, indicated at the *bottom*, are based on the phenotypic data tabulated on the *right*

**Fig. 3:**
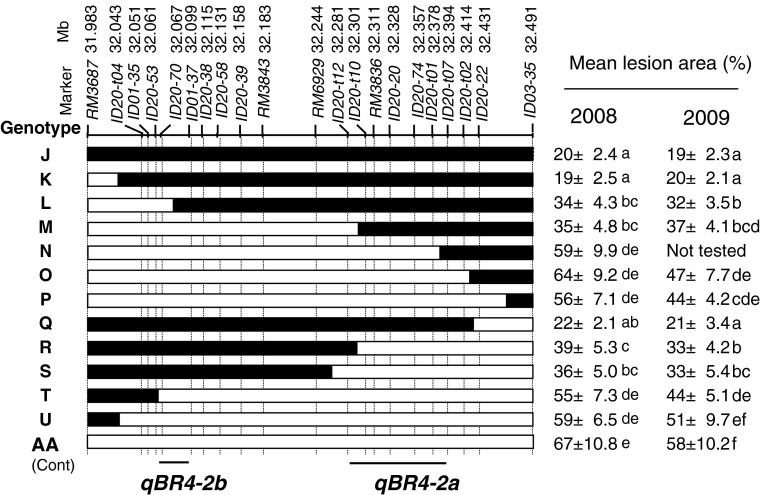
Physical map and graphical genotypes of sub-CSSLs around *qBR4*-*2a* and *qBR4*-*2b*. *Black bars* indicate regions derived from the resistant Owarihatamochi; *white bars* indicate those derived from the susceptible Aichiasahi. The locations of *qBR4*-*2a* and *qBR4*-*2b*, indicated at the *bottom*, are based on the phenotypic data tabulated on the *right*

The CSSL was further crossed with Aichiasahi to select recombinants within the substituted region in order to develop inbred lines having recombination within and around *qBR4*-*2*. First, 36 plants with independent recombination events were selected from 780 plants in the BC_4_F_2_ population. The position and size of the Owarihatamochi region in the recombinants were analyzed using an additional 7 restriction fragment length polymorphism (RFLP) and 14 simple sequence repeat (SSR) markers in the target region. The RFLP markers were obtained from the Nipponbare/Kasalath map (Kurata et al. 1994) and the SSR markers from previous studies (Akagi et al. 1996; IRGSP 2005; McCouch et al. 2002). DNA marker analysis followed our previous procedures (Fukuoka and Okuno 2001). Homozygous plants were selected from the progeny of recombinants, and self-pollinated progeny lines, called sub-CSSLs, were used for phenotyping. Sub-CSSLs were classified into eight genotypes B to I (Fig. 2). To dissect *qBR4*-*2*, we crossed a sub-CSSL with Aichiasahi to select recombinants within the 2.5-Mb region between the DNA marker loci *RM5503* and *RM348*. The DNA markers used are listed in Supplemental Table S1. These sub-CSSLs were classified into 12 genotypes J to U on the basis of DNA marker profiles (Fig. 3). We used a total of 30 sub-CSSLs selected from 3,103 individuals for mapping. As we detected three QTLs in the *qBR4*-*2* region, we crossed sub-CSSLs carrying one or two of the QTLs and selected lines having each combination of pairs or all three by means of marker-assisted selection in order to validate the effects of the combined QTLs.

### Assessment of blast resistance

In order to determine the map position of *qBR4*-*2*, we evaluated the blast resistance of homozygous progeny of the sub-CSSLs. We evaluated the resistance in an experimental field at the Aichi Agricultural Research Center (AARC), Mountainous Region Institute (Toyota, Aichi), or at the National Institute of Agrobiological Sciences (NIAS; Tsukuba, Ibaraki), where the disease pressure from blast fungus is particularly high and its progress is well monitored. The predominant fungal races are 007.0 in the former and 037.3 in the latter, and produced susceptible lesions on the plants.

Plants from 50 seeds per line were grown in 3 replications. The lesion area of 60- to 70-day-old plants was scored against a published reference scale (http://www.gene.affrc.go.jp/pdf/manual/micro-18.pdf, page 13). The susceptible recurrent parent Aichiasahi was grown on either side of each line.

### Data analysis

We constructed a genetic linkage map around the *qBR4*-*2* region using 192 F_2_ plants of the cross between the CSSL and Aichiasahi using Mapmaker software (Lander et al. 1987). The PROC GLM program of the Statistical Analysis Systems package (SAS Institute Inc.) was used to test differences in the phenotypic values among genotypes.

### Construction of bacterial artificial chromosome library, sequencing, and gene prediction

Megabase-size rice DNA was prepared from young leaves of Owarihatamochi as described (Zhang et al. 1995). A bacterial artificial chromosome (BAC) library was constructed by ligation of the megabase DNA with the pIndigoBAC vector (Epicenter) and transformation of BACs into *E. coli* DH10B cells (Invitrogen) (Osoegawa et al. 1998). The library consisted of 20,380 clones with an average insert size of 100 kb. The clones containing the *qBR4*-*2a* locus were screened using DNA markers *ID03*-*34* and *ID03*-*35*, and two positive clones (Owa28H01 and Owa28A18) were shotgun sequenced (Fleischmann et al. 1995). Putative coding sequences (CDSs) were predicted by the Rice Genome Automated Annotation System (RiceGAAS, http://ricegaas.dna.affrc.go.jp/; Sakata et al. 2002).

## Results

### Blast resistance in CSSL and sub-CSSLs

In the primary genetic analysis, we evaluated the CSSL and 36 sub-CSSLs representing nine genotypes (A to I) for blast resistance for 3 years in the field at the AARC (Fig. 2). The mean lesion area ranged from 13 to 62 % in 2004, from 11 to 50 % in 2005, and from 17 to 67 % in 2006. The lesion area of the donor Owarihatamochi was less than 1 % in all the tests. The mean lesion area of the CSSL (genotype A) was significantly smaller than that of the susceptible Aichiasahi (abbreviated as AA). Those of genotypes B and I were comparable to that of the CSSL. In contrast, genotype F had the largest mean lesion area among the sub-CSSLs, with the same level as AA. The rest (genotypes C, D, E, G, and H) were intermediate. This ranking was consistent across the 3 years. Both the distal (genotypes C, D and E) and proximal regions (G and H) of the donor segment on their own gave a significant decrease in mean lesion area compared with the AA. Therefore, we hypothesized that two regions, spanning marker intervals *RM3534*–*RM349* and *RM317*–*RM3217*, are associated with blast resistance. Furthermore, two genotypes carrying different lengths of the distal region (D and E) had small but significant differences in two of the 3 years. Therefore, we tentatively assigned two putative QTLs, *qBR4*-*2a* and *qBR4*-*2b*, in the marker interval *RM3534*–*RM349*. A QTL in the proximal region (*RM317*–*RM3217*), designated *qBR4*-*2c*, had a small effect that was significant only in 2004 (genotypes G and H).

### Delimitation of *qBR4*-*2a* and *qBR4*-*2b*

In order to validate and delimit *qBR4*-*2a* and *qBR4*-*2b*, we screened recombinants in a mapping population in which both loci segregated, and which lacked the resistance allele at *qBR4*-*2c*. We evaluated 30 sub-CSSLs representing 12 genotypes selected from 3,103 individuals in the field for 2 years. The mean lesion area of the sub-CSSLs ranged from 19 to 64 % in 2008 at the AARC and from 19 to 51 % in 2009 at the NIAS. The mean lesion area of the original sub-CSSL (genotype J) was significantly smaller than that of AA in both years, and two other genotypes (K and Q) were comparable to it (Fig. 3). In contrast, five genotypes (N, O, P, T, and U) had the largest mean lesion area among the sub-CSSLs. The rest (genotypes L, M, R, and S) were intermediate. The ranking was consistent across the 2 years. We concluded that this chromosomal region includes two loci: *qBR4*-*2a* (between markers *ID20*-*t12* and *ID20*-*t07*) and *qBR4*-*2b* (between *ID20*-*70* and *ID01*-*37*)*.* *qBR4*-*2a* had a slightly larger effect than *qBR4*-*2b.*

### Validation of *qBR4*-*2c* by QTL pyramiding

In order to validate the effect of *qBR4*-*2c*, we crossed a sub-CSSL carrying this QTL (genotype H) with one carrying *qBR4*-*2a* and *qBR4*-*2b* (D) and one carrying *qBR4*-*2a* only (E), and selected progeny homozygous for the resistance alleles at two or three loci. 2 years’ evaluation suggested that the addition of *qBR4*-*2c* significantly reduced the lesion area in the genetic background of genotypes D and E, and further confirmed the partial resistance conferred by genotype H (Table 1).

**Table 1:** Pyramiding of *qBR4*-*2a*, *qBR4*-*2b*, and *qBR4*-*2c* explains the effect of *qBR4*-*2*

Genotype	Expected resistance QTL^a^			Mean lesion area (%)^d^	
	*qBR4*-*2a*	*qBR4*-*2b*	*qBR4*-*2c*	2009	2010
A	1	1	1	11 ± 2.2a	13 ± 2.6a
D	1	1	0	23 ± 3.0b	25 ± 6.2b
E	1	0	0	31 ± 5.4c	45 ± 7.5c
H	0	0	1	41 ± 7.8d	46 ± 9.4c
D + H^b^	1	1	1	10 ± 2.4a	9 ± 2.0a
E + H^c^	1	0	1	22 ± 6.6b	31 ± 7.6b
AA(cont)	0	0	0	58 ± 6.8e	54 ± 8.4d

^a^ *1* Homozygous allele from resistant Owarihatamochi, *0* homozygous allele from susceptible Aichiasahi

^b^ *D* + *H* progeny lines obtained from the cross between D and H and selected by MAS

^c^ *E* + *H* progeny lines obtained from the cross between E and H and selected by MAS

^d ^Values followed by the same letter are not significantly different according to Tukey’s HSD test at 5%

### Candidate genes for *qBR4*-*2a* and *qBR4*-*2b*

**Fig. 4:**
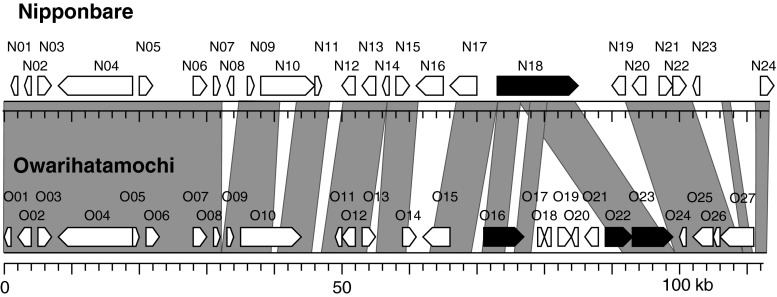
Sequence comparison of *qBR4*-*2a* between susceptible Nipponbare and resistant Owarihatamochi. Putative coding sequences (CDSs) are indicated by *boxes*; *black boxes* represent those that encode proteins with similarity to proteins containing a nucleotide-binding site (NBS) and leucine-rich repeats (LRRs). *Shading* indicates regions with very high sequence identity (>98% DNA identity overall) between genotypes. *Position zero* on the scale corresponds to 32,280,568 bp on the International Rice Genome Sequencing Project (IRGSP) build 5 pseudomolecules of the rice genome (Supp. Table S2)

**Fig. 5:**
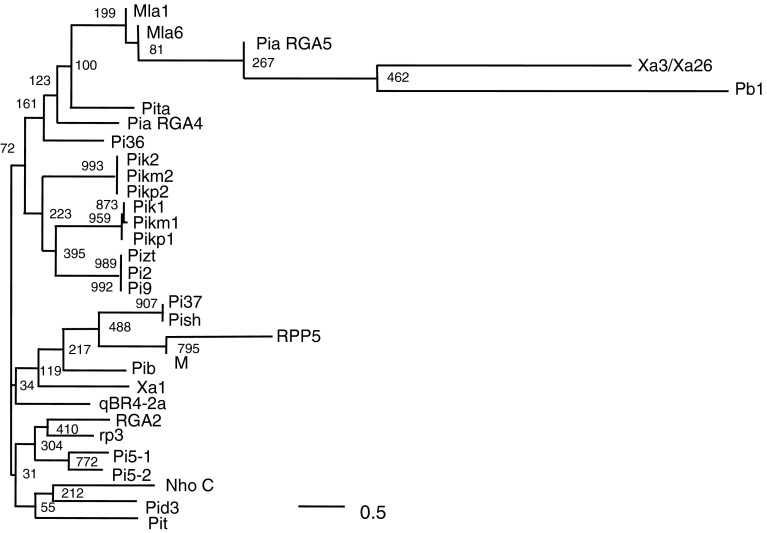
Phylogenetic analysis of the putative *qBR4*-*2a* with 30 other plant *R* genes. Deduced amino acid sequences of the putative nucleotide-binding site (NBS) site of *qBR4*-*2a* (O16) and of *R* genes obtained from GenBank were aligned, and a neighbor-joining phylogenetic tree was generated using CLUSTALW (http://clustalw.ddbj.nig.ac.jp/top-e.html) and Treeview software (http://taxonomy.zoology.gla.ac.uk/rod/treeview.html). *Numbers* on branches indicate the percentage of 1,000 bootstrap replicates which support the adjacent node. The unit branch length is 0.5 nucleotide substitutions per site (*bar*)

We delimited *qBR4*-*2a* and *qBR4*-*2b* to within regions of 113 and 32 kb, respectively, in the Nipponbare sequence (IRGSP 2005), where the Rice TOGO Browser (http://agri-trait.dna.affrc.go.jp/index.html; Nagamura et al. 2011) identified 24 and 2 putative CDSs (Supp. Table S2). The two putative CDSs in the *qBR4*-*2b* region show similarity to previously reported disease resistance proteins containing a nucleotide-binding site (NBS) and leucine-rich repeats (LRRs) (McHale et al. 2006), either or both of which are candidates for *qBR4*-*2b*. To sequence the *qBR4*-*2a* locus, we constructed a BAC library of Owarihatamochi and fully sequenced two clones, Owa28H01 and Owa28A18, containing *qBR4*-*2a*. Sequencing and RiceGAAS analysis revealed that the 113-kb sequence between markers *ID20*-*t12* and *ID20*-*t07* contains 27 putative CDSs (Supp. Table S3). Sequence comparison of the *qBR4*-*2a* region between susceptible Nipponbare and resistant Owarihatamochi revealed overall similarity, but several regions showed lower similarity owing to chromosomal reorganization (insertions or deletions and duplication) in the Owarihatamochi genome in a region containing putative CDSs with similarity to three genes encoding NBS-LRR disease resistance proteins (Fig. 4): CDSs O16, O22, and O23. O22 and O23 lack an NBS domain, leaving only O16 as the most probable candidate for *qBR4*-*2a*. A phylogenetic tree of deduced amino acid sequences of the NBS domain of O16 and of previously cloned disease resistance genes in rice and other crops obtained from public databases grouped O16 with a bacterial blight resistance gene of rice, *Xa1* (Yoshimura et al. 1998), and rice blast resistance genes *Pib*, *Pi37*, and *Pish* (Lin et al. 2007; Takahashi et al. 2010; Wang et al. 1999) (Fig. 5).

## Discussion

Recent progress in genomics has enhanced understanding of the genetic basis of agronomic traits, including those controlled by multiple loci in rice (Yamamoto et al. 2009; Yano and Sasaki 1997; Yonemaru et al. 2010). Yet, despite the great potential of marker-assisted selection in breeding programs, the use of beneficial QTLs from exotic germplasms is still a challenge.

One reason concerns the lower reliability of detection and the lower resolution of mapping of QTLs with minor effects in analysis using primary mapping populations, such as backcrossed inbred lines and recombinant inbred lines (Fukuoka et al. 2010). Advanced backcross progeny can be used to cope with this problem (Fukuoka et al. 2010), as we showed here by validating the effect of *qBR4*-*2* through the use of a CSSL. Importantly, our results suggest that *qBR4*-*2* is a gene complex comprising three loci, *qBR4*-*2a*, *qBR4*-*2b*, and *qBR4*-*2c,* which cumulatively enhance disease resistance. The effect of *qBR4*-*2c* was stably detected in the presence of the other two loci, although it was almost undetectable in the genetic background of the susceptible cultivar Aichiasahi. Such evidence partly explains the complicated genetic control of QTL-mediated resistance to blast (Rao et al. 2005; Wu et al. 2005). Understanding the isolate/race specificity of each individual QTL for blast resistance is crucial for deciding which resistance genes are to be used in rice breeding programs. Our methods show how such specificity can be identified.

Another reason concerns linkage drag, which is frequently observed in cross-breeding using exotic germplasms (Brown 2002; Ruge-Wehling et al. 2006), and which explains the difficulties in introducing QTL alleles for blast resistance into elite cultivars (Higashi 1995; Morimoto 1980; Saka 2006). Precise map information for a gene or QTL associated with traits of agricultural value is indispensable to marker-assisted elimination of undesirable traits tightly linked with the gene or QTL (Fukuoka et al. 2009). We determined the precise map location of two of the three QTLs detected here. Interestingly, these QTLs lie in the same region as a cluster of QTLs for traits that are strongly associated with productivity, including morphology and photosynthesis (Courtois et al. 2003; Ikeda et al. 2007; Saito et al. 2004; Sardesai et al. 2002; Takai et al. 2010). This observation highlights the importance of this region as a target for selection in breeding. Such findings will allow us to combine QTLs for blast resistance with QTLs for other agronomic traits while using marker-assisted selection to remove linkage drag.

Sequence comparison of *qBR4*-*2a* between Nipponbare and Owarihatamochi implies a complicated evolutionary history of this region, as suggested in other resistance gene complexes (Dixon et al. 1996; Wang et al. 1998; Xiao et al. 2001). The involvement of the *qBR4*-*2* region in blast resistance has been reported in several rice cultivars and wild relatives (Goto 1988; Hirabayashi et al. 2010; Miyamoto et al. 2001; Terashima et al. 2008; Wang et al. 1994; Xu et al. 2008). To determine whether the resistance is based on allelic differences at one or more of the *qBR4*-*2* loci is an important issue to clarify for the use of natural variation in blast resistance. The accumulation of rice genome sequence and haplotype information and the use of DNA genotyping technology will be helpful in clarifying this point (Ebana et al. 2010; Huang et al. 2010; Nagasaki et al. 2010; Yamamoto et al. 2010).

Our results suggest that allelic variation in one or more NBS-LRR genes is responsible for differences in blast resistance in rice. NBS-LRR genes are an important component in the evolution of plant resistance, mostly in race specificity (Bai et al. 2002; Bennetzen and Hulbert 1992; Hayashi and Yoshida 2009; Liu et al. 2007; Michelmore and Meyers 1998; Qu et al. 2006; Richter and Ronald 2000; Wei et al. 2002). Recent evidence suggests that tightly linked blast resistance genes epistatically control race-specific resistance at the *Pikm*, *Pi5*, and *Pia* loci (Ashikawa et al. 2008; Lee et al. 2009; Okuyama et al. 2011), but difficulties in validating quantitative differences have limited the number of reports on the additive effect of resistance genes. Our results confirm the effect of three loci in multiple trials and suggest that multiple QTLs contribute to the differences in the magnitude of effect.

NBS-LRR genes were not found in the *qBR4*-*2c* region in the Nipponbare genome. Sequence comparison between Nipponbare and Owarihatamochi and further fine genetic analysis of *qBR4*-*2c* will aid our understanding of QTL-mediated resistance to blast and the natural variation in the defense responses of plants. The characterization of lines combining QTLs, including the *qBR4*-*2* complex, will help us to better understand rice–blast interaction.

## Electronic supplementary material

Below is the link to the electronic supplementary material.

Supplementary material 1 (DOCX 24 kb)
