# Supplementary material for: A multiple gene complex on rice chromosome 4 is involved in durable resistance to rice blast
Source: Theor Appl Genet. 2012 Mar 25;125(3):551–9. doi: 10.1007/s00122-012-1852-4 (PMC3397134; doi:10.1007/s00122-012-1852-4)
Supplement: Supplementary file 1 — Supplementary material 1 (DOCX 24 kb) [file 122_2012_1852_MOESM1_ESM.docx]

**Supplemental Table S1**. Sequences of primers for the genetic markers used

| Primer | Forward (5'-3') | Reverse (5'-3') | Restriction enzymes |
| --- | --- | --- | --- |
| G264* | GCGTTGGTGTCAGCTAAGACT | TTGCACGTTCACAATTGTATATT | *Hin*dIII |
| OSR15 | CAACCAACGCCAAAAGCTAC | GTGGTACTCGCCCTGCATGA |  |
| C1016** | CAGTGAAGAAATGCCCAATG | ATTACAACCACCCCTCCTC | *Eco*RI |
| ID03-34 | CCGGAAACACTGTCGAGGTGC | GGCGTTCATCTCCTGCTTCCA |  |
| ID20-t04 | CGCGTGACTCTACTTCGGTGAA | CTCCGAGTTACATGCCCAACAG |  |
| ID01-35 | CCCTCGATGCGATGCGTGT | CCGCAAAGATGGAGACAGAAC |  |
| ID20-53 | TTTGGTTGGAGGGCTCGGTA | CCGGATCCACTCATTCAGCA |  |
| ID20-70 | TTAGGCTCCGTCAGGCTGCT | GCCTCTTCTTTCGGCCAACC |  |
| ID01-37 | TGCGAACCGAATAGCTGATGA | GGCGTGACCGTGTGAGCAC |  |
| ID20-38 | TCCTGCCTCCCTCCAAGATCA | GTGGTGACGGCACCTTATCC |  |
| ID20-58 | GCATAGGCGCCTCTTCCAAA | CCGAGAGCAGCTCGGTTGTT |  |
| ID20-39 | CGAAGATTGGAAATCCCTGCAT | TCATGCATAAGGCCACACATGA |  |
| ID20-t12 | GATTTGGGTGACGATTGACTTGTAC | TGTCCAACTTTAACTGTCCGTCTTA |  |
| ID20-t10 | TGTGACACCTACATAGTGTTGACCTAG | CATCGATCCAAGTCATCCAACTAC |  |
| ID20-20 | AATGGAACTTGGCGACCGATA | GCGGGTGCTACTGTCCAATAA |  |
| ID20-74 | GACAGGAGGATGGGGGCTCCAATC | GGAGAGAAACTTGGGGCA |  |
| ID20-t01 | GGGGAAGCATATACTTGTTTGAA | GCTGGCGTCTGGAGTATGTAGA |  |
| ID20-t07 | TAAGGACCAAAAAGAGAATAAGG | CTATCAAAATATACCAATAGCACAAA |  |
| ID20-t02 | AGATTACTGGTTAGCCTCTATGG | TTAAGGAAACATACTTAGCATGACCTA |  |
| ID20-22 | ATCACACCGCCGTATCATCATC | GCAGAGGGACGGCCAATATG |  |
| ID03-35 | GCTCCGGTGGCTCTTCGTG | AGGCTTAAGGCGAAAGGAAGT |  |

Map locations of marker loci are indicated in Fig. 2 and Fig. 3.

Markers are based on length polymorphism of PCR products unless otherwise noted.

*Cleaved Amplified Polymorphic Sequence (CAPS) marker.

** CAPS marker developed by the Rice Genome Research Program (RGP).

(http://rgp.dna.affrc.go.jp/E/publicdata/caps/index.html)

**Supplemental Table S2** Putative coding sequences (CDSs) predicted by RiceGAAS in the *qBR4-2a* region in the Nipponbare genome

| CDS No. | Proteins coded by predicted genes | Predicted length (bp) | Start (IRGPS build 5) | End (IRGPS build 5) |
| --- | --- | --- | --- | --- |
| N01 | Putative phosphatidate phosphatase LPIN3 | 281 | 32282133 | 32282414 |
| N02 | Putative LuxR-family transcriptional regulator | 549 | 32283494 | 32284043 |
| N03 | Putative 1-AGP acyltransferase | 1279 | 32285839 | 32287118 |
| N04 | Putative nucleolar pre-ribosomal-associated protein 1 | 12007 | 32288182 | 32300189 |
| N05 | Putative MAB2 | 1298 | 32301121 | 32302419 |
| N06 | Putative MAB2 | 1127 | 32308895 | 32310022 |
| N07 | Putative MAB2 | 1100 | 32311205 | 32312305 |
| N08 | Unknown protein similar to OSJNBb0060E08.4 | 455 | 32314159 | 32314614 |
| N09 | Putative MAB2 | 1121 | 32316812 | 32317933 |
| N10 | Putative RNA Binding Protein 45 | 8129 | 32318744 | 32326873 |
| N11 | Unknown protein similar to Os04g0625900 | 352 | 32327288 | 32327640 |
| N12 | Putative AT hook motif family protein | 2154 | 32330823 | 32332977 |
| N13 | Unknown protein | 1999 | 32333924 | 32335923 |
| N14 | Putative AT hook motif family protein | 623 | 32337680 | 32338303 |
| N15 | Unknown protein | 1817 | 32339442 | 32341259 |
| N16 | Putative mutator-like transposase | 3752 | 32342348 | 32346100 |
| N17 | Putative chloroplast lipocalin | 3406 | 32347767 | 32351173 |
| N18 | Putative NBS-LRR disease resistance protein homologue | 12629 | 32353617 | 32366246 |
| N19 | Unknown protein similar to OSJNBa0019K04.11 | 1193 | 32371999 | 32373192 |
| N20 | Putative F-box domain containing protein, expressed | 1054 | 32375222 | 32376276 |
| N21 | Putative transcription termination factor Rho | 2033 | 32377534 | 32379567 |
| N22 | Putative adenosylmethionine-8-amino-7-oxononanoate transaminase | 1643 | 32379926 | 32381569 |
| N23 | Putative polyprotein | 790 | 32383062 | 32383852 |
| N24 | Hypothetical protein similar to Epstein-Barr virus EBNA-1-like protein | 1043 | 32390966 | 32392009 |

**Supplemental Table S3** Putative coding sequences (CDSs) predicted by RiceGAAS in the *qBR4-2a* region in the Owarihatamochi genome

| CDSNo. | Proteins coded by predicted genes | Predicted length (bp) |
| --- | --- | --- |
| O01 | Putative LigA | 1344 |
| O02 | Putative low quality protein: polyketide synthase | 1303 |
| O03 | Putative 1-AGP acyltransferase | 1281 |
| O04 | Putative nucleolar pre-ribosomal-associated protein 1 | 12001 |
| O05 | Unknown protein | 396 |
| O06 | Putative MAB2 | 1302 |
| O07 | Putative MAB2 | 1128 |
| O08 | Putative MAB2 | 1101 |
| O09 | Putative MAB2 | 1113 |
| O10 | Putative RNA recognition motif family protein, expressed | 8767 |
| O11 | Hypothetical protein | 566 |
| O12 | Unknown protein | 1879 |
| O13 | Putative PIF-like transposase | 861 |
| O14 | Putative Mutator-like transposase | 1522 |
| O15 | Putative chloroplast lipocalin | 3408 |
| O16 | Putative NBS-LRR disease resistance protein homologue | 6591 |
| O17 | Unknown protein | 386 |
| O18 | Hypothetical protein | 1173 |
| O19 | Unknown protein similar to transposon protein, CACTA, En/Spm sub-class | 2509 |
| O20 | Hypothetical protein similar to ulp1 protease-like protein | 607 |
| O21 | Putative adenylate cyclase | 1887 |
| O22 | Putative NBS-LRR disease resistance protein homologue | 3159 |
| O23 | Putative NBS-LRR disease resistance protein homologue | 6385 |
| O24 | Putative YyaJ | 807 |
| O25 | Putative F-box domain containing protein, expressed | 3257 |
| O26 | Hypothetical protein | 118 |
| O27 | Putative F-box domain containing protein, expressed | 5319 |
